# Enhancing the use of stakeholder analysis for policy implementation research: towards a novel framing and operationalised measures

**DOI:** 10.1136/bmjgh-2020-002661

**Published:** 2020-11-06

**Authors:** Marysol Astrea Balane, Benjamin Palafox, Lia M Palileo-Villanueva, Martin McKee, Dina Balabanova

**Affiliations:** 1 College of Medicine, University of the Philippines Manila, Manila, Philippines; 2 Centre for Global Chronic Conditions, London School of Hygiene and Tropical Medicine, London, UK; 3 Department of Global Health & Development, London School of Hygiene & Tropical Medicine, London, UK

**Keywords:** health policy, public health

## Abstract

**Background:**

Policy is shaped and influenced by a diverse set of stakeholders at the global, national and local levels. While stakeholder analysis is a recognised practical tool to assess the positions and engagement of actors relevant to policy, few empirical studies provide details of how complex concepts such as power, interest and position are operationalised and assessed in these types of analyses. This study aims to address this gap by reviewing conceptual approaches underlying stakeholder analyses and by developing a framework that can be applied to policy implementation in low-and-middle income countries.

**Methods:**

The framework was developed through a three-step process: a scoping review, peer review by health policy experts and the conduct of an analysis using key informant interviews and a consensus building exercise. Four characteristics were selected for inclusion: levels of knowledge, interest, power and position of stakeholders related to the policy.

**Result:**

The framework development process highlighted the need to revisit how we assess the power of actors, a key issue in stakeholder analyses, and differentiate an actor’s potential power, based on resources, and whether they exercise it, based on the actions they take for or against a policy. Exploration of the intersections between characteristics of actors and their level of knowledge can determine interest, which in turn can affect stakeholder position on a policy, showing the importance of analysing these characteristics together. Both top-down and bottom-up approaches in implementation must also be incorporated in the analysis of policy actors, as there are differences in the type of knowledge, interest and sources of power among national, local and frontline stakeholders.

**Conclusion:**

The developed framework contributes to health policy research by offering a practical tool for analysing the characteristics of policy actors and tackling the intricacies of assessing complex concepts embedded in the conduct of stakeholder analyses.

**Key questions**

**What is already known?**

- Stakeholder analyses require assessment of the levels of power, position and interest of actors relative to health policies, but few empirical studies provide details of how the complex concepts embedded within these analyses are operationalised.

**What are the new findings?**

- There is no universally agreed way of conducting stakeholder analyses, as different studies followed diverse guidelines and frameworks and operationalised key concepts such as stakeholder power and interest in various ways.
- The developed framework proposes to assess stakeholder power as potential power in terms of access to resources, and position to reflect an actor’s actual exercise of power based on actions taken for or against a policy. Intersections between knowledge, interest, power and position must also be taken into account in stakeholder analyses.
- Both top-down and bottom-up approaches in policy implementation must be considered when analysing policy actors at the global, national and local levels.

**What do the new findings imply?**

- The developed framework addresses a gap in health policy research by offering a practical tool for analysing the characteristics of policy actors and by tackling the intricacies of assessing complex concepts while conducting stakeholder analyses.

## Introduction

Researchers seeking to influence policy must engage with relevant stakeholders. But whom and how? Stakeholder analysis can identify key actors in the policy process and develop strategies to engage with them.^1^ Stakeholders are defined by Varvasovszky and Brugha as “actors who have an interest in the issue under consideration, who are affected by the issue, or who – because of their position – have or could have an active or passive influence on the decision-making and implementation processes.”^2^

Stakeholder analysis can be used for different purposes in policy research, retrospectively to assess stakeholder roles in policy processes, or prospectively to inform future policy directions.^2^ It covers the entire policy cycle, from agenda setting to policy formulation, adoption, implementation and evaluation.^3^ Stakeholders with competing ideologies or interests can influence formulation of policies^4^ and can reshape adopted policies by contesting and negotiating their implementation.^5^

Stakeholders are typically analysed by their interests, position and, especially, their power.^6^ *Interest* refers to their concerns about how a particular policy will affect them^7^; *position* reflects their level of support for or opposition to the policy^1^; and *power* is their ability to affect policy, reflecting their resources and ability to mobilise them.^1^ While interest and position can be straightforward to ascertain, assessing power is more complex as it impacts on all steps of the policy process.^8^ Yet, power is often poorly characterised in empirical research on implementation of disease management policies, especially in low-and-middle income countries (LMICs).^9 10^

Sriram *et al* describe power as conceptually fluid, viewed on different levels, political angles and sociocultural lenses.^8^ In health policy, Lukes’ three faces of power include a first, visibly played out in the formal political arena; a second involving formal and informal processes underpinning development of political agendas; and a third, invisible but shaping the narrative on measures considered acceptable.^11^ Gaventa expands this approach, introducing the concept of a ‘power cube’ with one dimension represented as visible, hidden and invisible, as in Luke’s model; and a second categorising power as local, national and global. A third divides spaces for engagement into closed or decision-making by an elite group of actors; invited spaces that allow participation by citizens or beneficiaries; and claimed or created spaces emerging from social mobilisation or natural gatherings outside formal policy arenas. Gaventa argues that significant changes are possible by aligning strategies to axes, like a Rubik’s cube.^12^ VeneKlasen and Miller distinguish four expressions of power. ‘Power over’ is the capability of those who hold power to exert influence on those without, ‘power with’ involves synergy with different actors, ‘power to’ pertains to one’s own ability to act, while ‘power within’ refers to self-awareness and recognition of self-worth leading to action.^13^

In policy implementation, power relates to the distribution of authority in a system. The traditional top-down model sees actors deriving power from their place in a *de facto* hierarchy. Policies are formulated at national or international levels and cascaded downwards,^14^ those in higher tiers setting objectives to be accomplished by implementers.^10^ Bottom-up implementation focuses on the active role of implementers and their ability to modify or react to policies based on local context.^14^ It views implementation as interactive, involving negotiation and conflict.^14^

Stakeholder analyses face several challenges. Fast-changing policy environments can shift stakeholder positions so findings are time-bound.^15^ Having many potential stakeholders may pose difficulties, as does the ability to delineate personal and role-driven opinions of those in organisations. Other challenges include sensitivities around asking about power and interest, and potential bias arising from the position of the analysts, often immersed in the policy process themselves.^6^ These can be addressed using longitudinal studies that capture changing positions, limiting analyses to main stakeholders, capturing personal views and organisational positions, triangulating primary data against secondary sources, and self-awareness and diversity of analysts in research.^6^

Another challenge is that many existing analyses fail to fully describe the process by which power, position and interest are operationalised. Given the complexity involved, understanding how researchers assessed stakeholder characteristics becomes crucial to guide later stakeholder engagement and interventions. The process starts with defining concepts, choosing variables or domains to represent these concepts and operationalising them using relevant indicators. Where several questions measure a single concept, scales or indices can be composite measures.^16^

This paper contributes to the methodology of stakeholder analysis, especially the operationalisation of stakeholder characteristics in health policy implementation research. We reviewed conceptual approaches in stakeholder analyses, proposed an analytical framework that drew on those conceptual approaches and included domains and value scales used to operationalise stakeholder characteristics. The framework was reviewed by experts and field-tested by diverse policy actors involved in a study of hypertension in the Philippines.^17^ The field-testing of the framework with implementation of the Philippine Package of Essential Non-communicable Disease (NCD) Interventions, to identify and manage NCD risk factors in primary care,^18^ provides a rich backdrop for framework development, involving interaction with diverse policy actors within a pluralistic health system.

## Methods

### Scoping review

**Figure 1:**
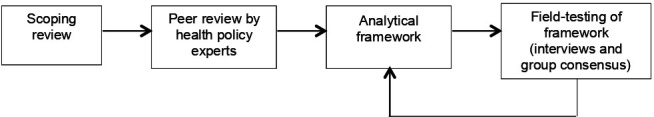
Methodology for framework development.

We performed several interlinked steps (figure 1). First, a scoping review of stakeholder analyses mapped key concepts and identified how stakeholder characteristics were operationalised, defined and measured in health research literature.

**Figure 2:**
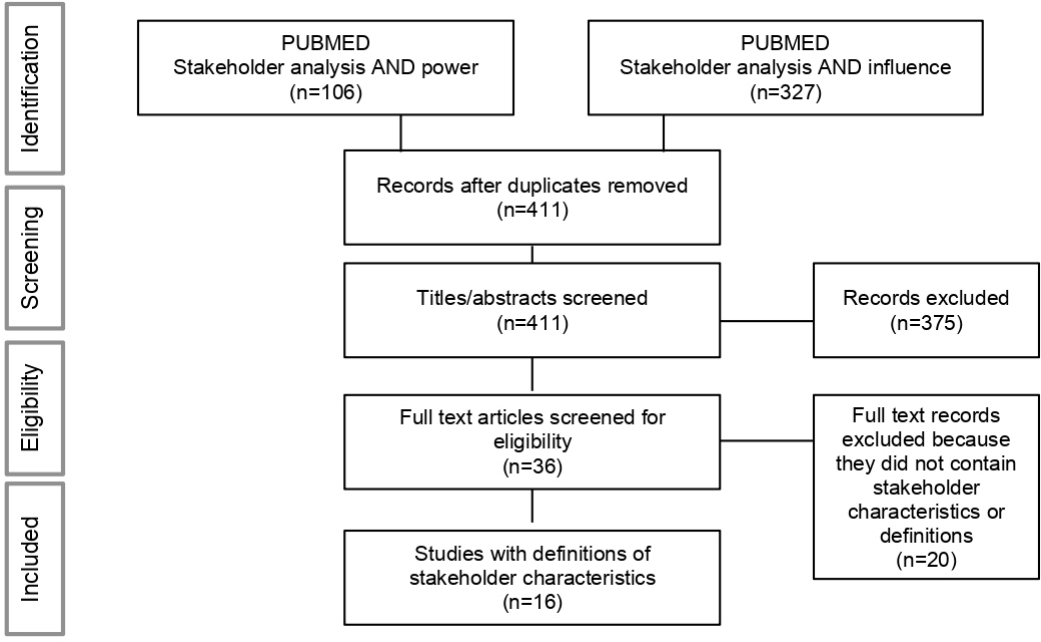
Flow diagram of scoping review on stakeholder characteristics.

Our scoping review process was guided by Arksey and O’Malley’s framework, with five stages: identifying the research question, identifying relevant studies, study selection, data charting and collating, and reporting results.^19^ We followed the Preferred Reporting Items of Systematic Reviews and Meta-Analysis extension for Scoping Reviews (PRISMA-ScR) (figure 2 and online supplemental appendix 1).^20^ Papers between January 2009 and July 2019 that included definitions of stakeholder characteristics in the analysis were eligible. Studies that did not provide definitions of characteristics were excluded.

10.1136/bmjgh-2020-002661.supp1 Supplementary data

Characteristics of power and influence were central to the analytical process. PubMed was searched with the key words ‘Stakeholder analysis AND power’ and ‘Stakeholder analysis AND influence’ which together generated 433 records. Duplicates were filtered using EndNote V.X9, leaving 411 unique records. A researcher screened titles and abstracts for eligibility. Sixteen were deemed relevant and analysed (online supplemental appendix 2). A manual search of selected references gathered relevant guidelines on methodologies for operationalising stakeholder characteristics, and were reviewed as part of the framework development.

Our data charting form extracted the following from each paper: year of publication, authors, study location, purpose of analysis, framework and guidelines used, definitions of stakeholder characteristics, and domains used to assess characteristics. Using a narrative synthesis approach, the scoping review generated an initial framework synthesising definitions and domains of stakeholder characteristics such as knowledge, interest, position and power, and methods used to assess them.

### Review by experts

The initial framework was sent for peer-review by international experts on health policy and health systems leadership, purposively selected due to (1) their expertise in conducting policy and stakeholder analyses in LMICs, (2) self-identifying as policy analysts and applying their skills in different projects, and (3) work highly regarded by peers or frequently cited. We initially contacted five via email, with three providing detailed reviews. They were from academia with doctoral degrees in health policy and politics and at least 10 years of experience in health policy and systems research. Specifically, the experts were asked to comment on the definitions, domains and scales used to assess stakeholder characteristics related to knowledge, power, interest and position. This was followed by a review of literature provided by experts, who guided us to particular theories and empirical work on power,^11 12 21 22^ which we supplemented with a manual search of selected references. We included references based on their fit with our study and revised our framework based on expert comments and the literature.^5 8 23^

### Field-testing of the framework in the Philippine context

We field-tested the revised framework in a study on implementation of an NCD policy in the Philippines to ascertain its appropriateness for assessing the levels of knowledge, interest, position and power of stakeholders. Field-testing was via key informant interviews from August 2019 to March 2020, and a 1-day consensus workshop in November 2019.

Eighteen key informants were selected via purposive sampling, which were identified either through document review or a snowball technique wherein respondents were requested to identify stakeholders with whom they usually collaborate. Identified stakeholders were eligible for interview based on their engagement with policy implementation and views of other stakeholders. This technique was particularly useful in narrowing down interviewees as it identified those currently wielding power in implementation processes, and those that can potentially influence other implementers.

A semistructured interview topic guide was developed based on the framework (online supplemental appendix 3). The interviews took between 30 and 90 min and were conducted in a mix of Tagalog and English depending on the preference of the interviewee. Written consent forms were requested, and participant numbers were assigned to interviewees to protect their identities.

A 1-day consensus-building workshop was conducted to further field-test the framework by assessing key stakeholders according to their level of knowledge, interest, power and position, and to evaluate the framework’s performance. Nine stakeholders from academic institutions, non-government organisations, professional societies, local governments and frontline health workers participated in the consensus workshop and engaged in discussions (table 1).

**Table 1:** Organisations that participated in the stakeholder analysis study

Interview respondents		Consensus workshop participants	
**Organisation**	**No**	**Organisation**	**No**
Department of Health (Central and Regional)	5	Academia	2
Local government units	3	Local government unit	3
Professional society	2	Frontline health worker	2
Multilateral organisation	4	International organisation	1
International and national NGOs	2	Professional society	1
Academia	1		
Media	1		

NGOs, non-government organisations.

Given the varied experiences and perspectives, and the potential professional hierarchies in the assembled group, the workshop was designed to solicit expert views using elements of focus group discussion to allow participants and facilitators to freely share knowledge and evidence and discuss each topic; and elements of the Delphi process, allowing participants to provide anonymous input.^24–27^ The workshop sought to achieve consensus on four topics: (1) identification of key stakeholder groups; (2) understanding of definitions of framework concepts, domains, indicators and scoring; (3) scoring based on stakeholder characteristics as defined in the analytical framework; and (4) determining strategies on engaging organisations identified as important and influential in implementation.

From an initial long list of stakeholders, participants narrowed the list down to 14 key actors. Following a framework orientation presentation, participants then scored the key actors on a scale of 0–3 based on their level of knowledge, interest and power; while stakeholder position was scored 1–5 based on the strength of support for the policy. After a first round of scoring, a summary was shown to the participants and a facilitated discussion ensued which clarified participants’ understanding and achieved consensus on the definitions of framework concepts, domains, indicators and scores, and to gather information on their experiences of assessing levels of stakeholder characteristics in a Delphi exercise. This was followed by further focus group discussion to achieve consensus on the scoring of each key stakeholder’s level of knowledge, interest, power and position. Our general consensus processes used a conventional group problem-solving approach involving (1) problem clarification, (2) agreement on deliberation procedures, (3) information and perspective sharing, (4) option development, (5) group selection of the preferred option,^26^ which was operationalised using simple majority vote. Key points from the discussion were recorded by the research team and integrated into the final version of the framework. A power-position matrix was then shown to the participants based on the results and a consensus-oriented focus group discussion ensued to determine strategies for engaging with stakeholders based on their location in the matrix.

Key informant interviews and the consensus building workshop were recorded, with audio files transferred to an encrypted laptop and transcribed verbatim. Feedback from the interviews and consensus building exercise were then taken into account in the revision of the framework to ensure its appropriateness in analysing stakeholders in the Philippine context and the topic area (CVD). The framework was reviewed and revised at every step by the researchers who were health professionals and/or faculty members from well-known universities.

## Results

### Scoping review

Sixteen articles were analysed, of which 12 applied to policy processes, 2 to health interventions, 1 was set in an organisation and 1 was a methodological paper. There was a mix of 9 prospective analyses and 7 retrospective analyses included in the review. In the 15 empirical studies included, analyses were applied across different contexts with 3 studies conducted in low-income economies, 4 in lower-middle income economies, 4 in upper-middle income economies and 4 in high-income economies.

The review revealed the absence of a standard way of operationalising characteristics in a stakeholder analysis, with different empirical studies over the past decade applying a variety of frameworks and guidelines. Some studies followed a single guideline when analysing stakeholder characteristics. Table 2 presents an overview of the different frameworks, including the stakeholder characteristics assessed in each study. Among those that followed a single framework, most used Schmeer’s guidelines, an approach recommended by WHO,^1^ which proposed to analyse stakeholders based on seven characteristics. Studies that adapted this, however, selected anywhere from 2 to 7 of the proposed characteristics, depending on which were deemed appropriate to the context being analysed.^28–31^ Six of the 16 papers did not follow a particular guideline, but drew on several sources to define and operationalise stakeholder characteristics. An example of this is Abiiro and McIntyre’s study on the premium payment policy in Ghana which adapted definitions of characteristics from various methodological papers and studies, and determined domains of power from stakeholder insights.^32^

**Table 2:** Approaches and characteristics used in the stakeholder analysis studies

Source	Stakeholder characteristics used in studies	Studies (n)
Adapted from Schmeer’s Stakeholder Analysis Guidelines^1^	Power Interest Position Leadership Knowledge Alliances Attitude	4^28–31^
Adapted from methodology papers by Varvasovzky and Brugha^2 42^	Position Influence	1^38^
Stakeholder Salience Theory^43^	Power Legitimacy Urgency	1^44^
Contextual Interaction Theory^45^	Power Motivation Information	1^46^
Combination of different guidelines and previous studies	Understanding Interest Power Position Influence	6^6 32–34 47 48^
Developed from stakeholder views	Power	1^36^
No specific guidelines or approach mentioned	Power Interest Position Influence	2^35 39^

When defining stakeholder characteristics, power and influence were used interchangeably in several studies and were defined, variously, as the ability of a stakeholder to affect policy formulation or implementation, their access to resources and ability to mobilise them.^6 33–35^ Three studies defined power as the stakeholder’s ability to influence policy, or assessed power and influence separately.^29 32 36^

Interest was defined in terms of the advantages or disadvantages that policy implementation conferred on a stakeholder, or their political stake or degree of involvement in an issue.^1 37^ The definition of position was more or less consistent across different studies, and indicates the level of support or opposition towards a policy or a programme.

There seems to be a general agreement across studies on the definitions of power, interest and position, suggesting that there is an agreement at the conceptual level. There is, however, considerable variability in how these characteristics were operationalised in guidelines and empirical work.

Table 3 shows domains that have been proposed in guidelines and methodological papers to assess stakeholder characteristics as well as domains that have been applied in empirical studies. We examined five guidelines and frameworks cited in the studies shown in table 2, and one methodological paper identified in our search. Out of these six, we included four that provided domains for assessing power, interest or position in the table.

**Table 3:** Domains used in stakeholder analysis guidelines, methodological papers and studies to assess power and influence, interest, and position

Domains for characteristics	No of guidelines/method papers (N=4)	No of empirical studies (N=7)
***Domains for power or influence***		
Technical/professional knowledge/skills	1^47^	3^28 32 36^
Decision-making		3^28 33 38^
Political/influential position	2^1 47^	2 28 36
Financial power/money	3^1 43 47^	2 28 36
Legal mandate	1^47^	2^28 32^
Human	1^1^	
Technological	1^1^	
Ability to place the issue on the agenda		2^33 38^
Legislative power for policy approval		2^32 38^
Influence over policy outcomes		2^32 38^
Attribution of power (actor’s power as perceived by themselves and others)	1^49^	
Ability to mobilise on the issue	1^1^	2^35 38^
Coercive: physical resources of force, violence or restraint	2^43 47^	
Normative: symbolic influences	1^43^	
Connections to influential stakeholders	1^47^	
Ownership/control of resources	1^49^	2^32 35^
Voting power/influence over voters		1^32^
Involvement in policy formulation		1^32^
Willingness to engage in policy discussions		1^39^
Ability to be heard in discussions		1^39^
Ability to influence other actors		1^39^
Ability to influence public opinion		1^32^
Directly or indirectly take action for or against the policy		1^35^
Control over implementation at the local level		1^32^
Determine policy success and sustainability		1^32^
Possession of privileges		1^35^
Ability to organise members		1^32^
No of votes	1^47^	
***Domains for interest***		
Overall perceived impact	1^1^	1^32^
Key interest/concerns	1^1^	1^32^
Professional affiliation		1^33^
Stakeholder agendas		1^33^
Status within the community		1^33^
Pursue benefits for stakeholder	1^47^	
Achieve equitable treatment for player’s group	1^47^	
Advance player’s view of common good	1^47^	
Garner more resources	1^47^	
Preserve power	1^47^	
***Domains for position***		
Level of support or opposition	2^1 47^	6^28 32 33 35 38 39^

For studies that conducted analyses in specific policy contexts, only 7 out of 15 papers provided details on what specific aspects of stakeholder characteristics were evaluated. Among the seven papers that included such details, six conducted prospective analyses with stakeholders at the agenda setting,^36 38^ policy formulation^32 33 39^ and intervention planning stages.^35^ One paper conducted a retrospective analysis applied to policy implementation.^28^

It should be noted that while the guidelines offered well-defined characteristics, some offered flexibility for researchers to use their own assessment criteria depending on their aim or specific contexts. Even in studies that proposed domains, some of these were defined broadly, such as ownership or access to resources, which still allows for researchers to ultimately define which particular resources to assess. Examination of empirical studies, therefore, provides helpful insights on which domains can be applied to actual policy contexts. Table 3 reflects common domains used for power or influence, interest and position revealed by our review.

Based on the scoping review findings, four characteristics were selected for inclusion in our adapted framework: knowledge, interest, power and position. Power, interest and position were the most frequently assessed characteristics in the stakeholder analysis literature, while knowledge was included in recognition that a stakeholder’s understanding of a policy may determine their level of interest and perception of how it can potentially affect them.^32^

Aside from the results of the scoping review, a methodological paper and several studies informed the development of the initial analytical framework; these include Schmeer’s definitions of characteristics, value scales from Caniato *et al*, and domains to assess power from Abiiro and McIntyre’s study.^1 30 32^ Table 4 shows a summarised version of the initial stakeholder analysis framework developed for policy implementation research and the subsequent modifications made after the expert review, key informant interviews and the consensus building exercise.

**Table 4:** Summary of stakeholder characteristics, definitions, domains and value scales in the initial framework, and key changes made after expert review, key informant interviews and consensus building exercise

Initial framework based on scoping review	Key changes after expert review	Key changes after key informant interviews and consensus building exercise
**Knowledge**		
**Definition:** Stakeholder’s level of knowledge and understanding of the policy **Domains:** Knowledge of policy (awareness and ability to describe key features) Understanding of policy purpose **Value scales:** 1—No or minimum knowledge, 2—General knowledge, 3—Extensive knowledge	**Definition:** Retained **Changes in domains:** Knowledge of policy and its implementation Source of information (added) **Value scales:** Retained	**Definition:** Retained **Changes in domains:** Operational knowledge of policy Awareness of policy (added) **Changes in value scales:** 0—No knowledge, 1—Limited knowledge, 2—General knowledge, 3—Extensive knowledge Note: General knowledge defined as operational knowledge on policy, while extensive knowledge includes both operational knowledge and understanding of policy rationale
**Interest**		
**Definition:** Extent to which stakeholders perceive policy implementation as relevant and likely to affect them **Domains:** Relevance of policy to stakeholder Willingness to participate in implementation Likelihood to affect stakeholder **Value scales:** 1—No or minimum interest, 2—Limited interest, 3—General interest, 4—High interest	**Changes in definition:** Stakeholder’s motivations and perceived impact of policy implementation to their own organisation **Changes in domains:** Policy objective core to organisation’s mission Policy is a priority for organisation Perceived impact of policy implementation to own organisation **Value scales:** Retained	**Definition:** Retained **Changes in domains:** Perceived policy impact in terms of opportunities and costs to the stakeholder **Changes in value scales:** 0—No Interest, 1—Limited interest, 2—General interest, 3—High interest
**Power**		
**Definition:** The ability of the stakeholder to affect policy implementation **Domains:** Capacity to design policies Capacity to fund policy implementation Capacity to implement policy Ability to lead and gather support from stakeholders Ability to influence public opinion **Value scales:** 1—Low, 2—Medium, 3—High Note: Stakeholders rated based on possession and control of resources	**Definition:** Retained **Changes in domains:** ***Political authority*** (a) Direct: Derived from hierarchy, legal mandate, regulatory regimes. (b) Indirect: Ability to create incentives and constraints for other act. ***Financial capacity*** Possession and control of financial resources ***Technical expertise*** Technical capacity to produce, interpret and disseminate knowledge and information. ***Leadership*** Ability to build partnerships and motivate other stakeholders for or against policy implementation. **Changes in value scales:** 1—Low, 2—Medium, 3—High Note: Stakeholders rated based on possession and control of resources and ability to make decisions in policy implementation	**Changes in definition:** The potential ability of the stakeholder to affect policy implementation based on resources **Changes in domains:** ***Leadership*** (a)Ability to build partnerships, motivate other stakeholders and/or shape opinion for or against policy implementation. (b)Personal attributes of individuals within the organisation which can include charismatic authority, personal commitment and motivation **Changes in value scales:** 1—Low, 2—Medium, 3—High Note: Stakeholders rated based on possession and control of resources and potential to affect policy implementation
**Position**		
**Definition:** Whether the stakeholder supports, opposes or is neutral about policy implementation **Domains** Degree of support or opposition to policy **Value scales:** 1—Opponent, 2—Moderate opponent, 3—Neutral, 4—Moderate supporter, 5—Supporter	**Definition:** Retained **Changes in domains:** Actions taken to demonstrate support or opposition to policy (added) **Value scales:** Retained	**Definition:** Retained **Changes in domains:** Degree of support or opposition to policy expressed through use of potential power or resources Actions taken to demonstrate support or opposition to policy **Changes in value scales:** 1—Strong opponent, 2—Moderate opponent, 3—Neutral, 4—Moderate support, 5—Strong support

Source: Definitions, domains and value scales for the frameworks were adapted from elements in the methodological papers and studies of Varvasovsky and Brugha (2000), Schmeer (2000), Abiiro and McIntyre (2013), Lehmann and Gilson (2013), Caniato *et al* (2014), Dalglish *et al* (2015) and Sriram *et al* (2018) and feedback from health policy experts and stakeholders.

### Feedback from expert review

The health policy experts provided feedback on the definitions of stakeholder characteristics and the domains used to assess them in the initial version of the framework. For knowledge, one expert suggested that the domains and value scales included seemed to imply that the policy is clearly laid out when, in reality, policies can be vague or not publicised. To address this comment, source of information was added as a domain in the framework to identify gatekeepers and determine the process and potential gaps in the transfer of knowledge.

With regard to interest, an expert argued that the concept refers to the concerns and driving motivations of stakeholders and how policies impact their organisation. Based on this feedback, the definition of interest was revised and domains were changed to reflect whether the policy is considered as a priority or perceived to affect the stakeholder in any way.

All three experts provided comments on the characteristic of power and suggested a review of relevant theories and empirical work analysing power to see how these can be incorporated in the framework. Following this feedback, additional literature on power was reviewed to determine its different dimensions and how these can be assessed in practice. A study by Dalglish *et al* applied to the policy for integrated community case management of childhood illness in Niger was particularly relevant as it examined a range of power theories and opted to select three dimensions of power deemed to be relevant to the country’s context: political authority, financial resources and technical expertise.^23^ These three domains also emerged in the scoping review and were thus incorporated in the revised analytical framework to be field-tested in the Philippine setting.

Still pertaining to power, one of the experts commented on how the domains seemed to be underpinned by a top-down view of implementation and may not take into account bottom-up approaches. Accordingly, the domain of ‘Leadership’ was added to describe a stakeholder’s ability to convene partners and mobilise them to work together to implement a policy. The additional domain was drawn from Lehmann and Gilson’s study on the micro-practices of power of community health workers in South Africa.^5^

Finally, one expert highlighted how position and interest are linked, as a stakeholder’s perception of how policy will impact their organisation can affect their level of support. As a result, another domain, ‘actions taken to demonstrate support or opposition’, was added to the framework to draw out ways in which stakeholders express their positions during policy implementation.

### Field-testing the appropriateness of the framework to CVD policy in the Philippines

Overall, the framework was found to be acceptable and appropriate to the policy context in the Philippines during the interviews and consensus building exercise. There were clarifications and discussions, however, about some of the characteristics and on how to evaluate levels of knowledge, interest, power and position of stakeholders, which led to further refinement of the framework.

With regard to knowledge, participants in the group consensus exercise differentiated between operational knowledge of the policy and understanding the overall policy goal, as some stakeholders may know how to implement the policy’s components, without necessarily being aware of what the policy seeks ultimately to achieve. Following discussions, four domains were included in the final framework to reflect awareness of policy, operational knowledge of policy, understanding of policy rationale and source of information. Value scales were likewise revised to categorise extensive knowledge as understanding both policy rationale and implementation issues, general knowledge as operational know-how in implementation, while limited knowledge refers to awareness about the policy without knowing specific details about it.

Another point of discussion during the group exercise was the link between awareness and interest, and how knowledge of the policy can determine level of interest. Those unaware of the policy may thus appear to have low interest, highlighting the need to assess interest in conjunction with knowledge. Interview findings suggest that asking about interest can also be potentially sensitive as it delves into the underlying motivations of different stakeholders. The direct question about policy impact on the organisation seemed to be unclear for some stakeholders and follow-up questions exploring opportunities and costs of the policy, as well as providing examples, helped stakeholders think more concretely about it. In the framework, this translates into specifying perceived impact as opportunities and costs to the stakeholder.

An important issue tackled during the consensus workshop was whether to rate stakeholders based on their potential power or actual exercise of power. A stakeholder, for example, may have resources and, as such, the potential to be involved in implementation, but for one reason or another does not fully exercise their potential power. To resolve the issue, the participants reached an agreement to rate the power characteristic as potential power, and to reflect the actual exercise of power when rating overall position.

A final issue on the link between personal attributes and stakeholder power arose during the interview process. Within organisations there may be charismatic and motivated individuals that help move policy implementation forward. Thus, personal attributes were included in the final framework under the domain of leadership to reflect the role of individuals in policy implementation. Table 5 shows the final revised framework based on combined feedback from expert consultations, interviews and group consensus exercise.

**Table 5:** Finalised framework for stakeholder analysis applied to the PhilPEN policy implementation context

**Knowledge**	
**Definition:** Stakeholders’ knowledge and understanding of the policy **Domains:** Awareness of policy Operational knowledge of policy Understanding of policy rationale Source of information	**Value scales:** 0—No knowledge Stakeholder is not aware of policy 1—Limited knowledge Stakeholder is aware of policy but have minimal knowledge about its purpose or implementation 2—General knowledge Stakeholder has operational knowledge about policy 3—Extensive knowledge Stakeholder understands policy rationale and has operational knowledge of policy
**Interest**	
**Definition:** Stakeholder’s motivations and perceived impact of policy implementation to their own organisation. **Domains:** CVD control core to organisation’s mission Policy is a priority for organisation Perceived policy impact in terms of opportunities and costs to the stakeholder	**Value scales:** 0—No interest Policy is not considered a priority and not perceived to impact stakeholder 1—Limited interest Policy is not considered a priority and has minimum impact on stakeholder 2—General interest Policy is a priority and has moderate impact on stakeholder 3—High interest Policy is part of the stakeholder’s core mission and has high perceived impact on stakeholder
**Power**	
**Definition:** The potential ability of the stakeholder to affect policy implementation **Domains:** ***Political authority*** Direct: Derived from hierarchy, legal mandate, regulatory regimes. Indirect: Ability to create incentives and constraints for other actors. ***Financial capacity*** Possession and control of financial resources ***Technical expertise*** Technical capacity to produce, intrepet and disseminate knowledge and information ***Leadership*** Ability to build partnerships, motivate other stakeholders and/or shape opinion for or against policy implementation. Personal attributes of individuals within the organisation which can include charismatic authority, personal commitment and motivation.	**Value scales:** 1—Low power Stakeholder possesses and has control over use of one to two sources of power, low potential to affect policy implementation 2—Medium power Stakeholder possesses and has control over use of two to three sources of power, has moderate potential to affect policy implementation 3—High power Stakeholder possesses and has control over use of three to four sources of power, has high potential to affect policy implementation
**Position**	
**Definition:** Whether the stakeholder supports, opposes or is neutral about policy implementation **Domains:** Degree of support or opposition to policy expressed through use of potential power (sources of power) Actions taken to demonstrate support or opposition to policy	**Value scales:** 1—Strong opponent Stakeholder uses potential power to strongly act against policy implementation 2—Moderate opponent Stakeholder uses potential power to moderately act against policy implementation 3—Neutral Stakeholder does not use potential power and does not act for or against policy implementation 4—Moderate support Stakeholder uses potential power to moderately act in support of policy implementation 5—Strong support Stakeholder uses potential power to act strongly in support of policy implementation

Source: Definitions, domains and value scales for the framework were adapted from elements in the methodological papers and studies of Varvasovsky and Brugha (2000), Schmeer (2000), Abiiro and McIntyre (2013), Lehmann and Gilson (2013), Caniato *et al* (2014), Dalglish *et al* (2015) and Sriram *et al* (2018) and feedback from health policy experts and stakeholders.

## Discussion

In this study, we present an adapted framework for stakeholder analysis that draws on empirical research, theory and advice from health policy experts, specifically developed for application to health policy contexts in LMICs. Its key domains and characteristics are fully operationalised. From our experience of taking a flexible and iterative approach to develop and field-test the framework in the Philippines, we believe it is a practical tool that is able to assess the stakeholder landscape in which a health policy is implemented and examine complex stakeholder characteristics in a rigorous, transparent, yet straightforward manner—a process that is seldom described well in empirical stakeholder analyses.^6^

While existing guidelines and frameworks for stakeholder analyses clearly define stakeholder characteristics, the process of operationalising or measuring these concepts is often left to the discretion of researchers to ensure they are fit for purpose and are adapted to their particular context. It is, however, important that empirical studies explicitly state the criteria for assessing characteristics to minimise bias,^2^ reduce ambiguity and allow the analysis to be replicated by other scholars intending to do similar studies. Our study contributes to the stakeholder analysis literature by describing our process, and the intricacies of identifying domains to include and putting a value on abstract concepts. This is a critical step required in analyses, but posing challenges that are not discussed adequately in the literature. By synthesising the most frequently used domains in studies, bringing insights from studies on power of actors outside of stakeholder analyses and going through an iterative process of operationalisation, the study offers a framework that can more easily be adapted and applied by other researchers. The framework also contributes to the overall discussion on power of actors and how to assess this, especially at the policy implementation stage.

The development process identified multiple intersections between stakeholder characteristics. Level of knowledge was linked to level of interest, as stakeholders unaware of the policy may be perceived as having low interest in its implementation, suggesting the need to analyse interest in conjunction with knowledge. This finding is consistent with Abiiro and McIntyre’s study, which postulated that a stakeholder’s understanding of a policy affects its perceived impact or interest in it.^32^ Interest was also linked to position, as the perceived impact of the policy on the stakeholder determines whether or not they will support or oppose its implementation. This finding is consistent with the definition of interest in other studies as ‘positive and negative impact’^40^ or ‘advantages and disadvantages’ of the policy to the stakeholders.^1^

The link between power and position was also explored, as the question of whether to measure potential power based on resources versus actual exercise of power determined through stakeholder actions became pertinent. Discussions revealed that there is value in looking at these two separately in order to identify stakeholders with high potential power, but who are not fully exercising this power in their implementation efforts. Such differentiation is also helpful when determining appropriate stakeholder engagement strategies. As a result, power, as used in the framework, meant potential power, while position reflected the exercise of power in terms of the actual use of available resources and actions taken by the stakeholder to support or oppose policy implementation.

The operationalisation process highlighted the difficulty of assessing stakeholder power. With the abundance of theories on power and its implicit and explicit manifestations in stakeholder interactions,^8^ it was challenging to determine which particular domains to include in the framework. Domains of power found in the stakeholder analysis literature typically identify access to sources of power, but discussions with stakeholders revealed that access to these sources is only one aspect of power, and effective use of potential power to achieve policy outcomes is also key but more challenging to assess. Evaluating the exercise of power by different actors in a stakeholder analysis involves the examination of policy actors interacting at the global, national and local levels. At the international level, some studies have shown that donors can control implementation outcomes through conditions stipulated in funding agreements^41^ and can also influence different stages of the policy process through leverage of financial resources, technical expertise and intersectoral pressure.^21^ Frontline providers, on the other hand, can exercise ‘micro-practices of power’ through day-to-day decision-making that can either support or subvert intended policy outcomes.^5^ Therefore, a comprehensive assessment of power requires sampling that provides sufficient representation of perspectives from global down to local levels. This task becomes even more complex and onerous in highly heterogeneous settings, which may result from factors, such as health system decentralisation, as in the Philippines.

Furthermore, when analysing stakeholders involved in policy implementation, it is important to consider incorporating both top-down and bottom-up approaches to account for the important role that frontline workers play in the implementation process. Implementing actors, often considered as having low power, can actually exercise very high levels of discretionary power (eg, by withholding labour), which, when done as a group, can undermine a policy’s goals.^5^

The new framework has several limitations. Since it was applied in the context of health policy implementation in an LMIC context, discussions were mostly focused on assessing characteristics of actors in implementing the policy as opposed to their ability to advocate or design policies, which would be more relevant at the policy formulation stage. While the domains can also be seen as relevant for assessing actors across different policy stages, high-income economy settings or different fields outside health policy research, its application to these contexts is beyond the scope of the study.

Although the study touched on the concept of power, it focused more on practical domains for assessing power among stakeholders, which was mainly sourced from previous empirical studies and feedback from experts and stakeholders. Domains of power identified in the framework were drawn mostly from more visible sources of power, or those that can be verified through document review, interviews and consensus among stakeholders. While the results of the interviews can provide some insights on the less visible forms of power at play during implementation, an additional layer of analysis may be needed to situate power dynamics between actors within the broader macro-political context and societal structures, such as those along the lines of gender, class or race.^10^ An example of this process of contextualisation can be seen in Gilson *et al*’s study in South Africa and Tanzania which identified situational, structural, exogenous and cultural factors affecting stakeholder interactions in universal health coverage debates.^6^ Sources of power should also be treated as context dependent and time bound as the power of stakeholders may shift over time and may only be applicable in certain contexts.^10^

Another limitation is that the scoping review only used one database, PubMed. While we reviewed additional literature provided to us by the experts we consulted, allowing us to draw insights from other relevant fields such as political science, it was beyond the scope of the project to attempt a comprehensive review of literature from all the fields that might have something to say. We felt given the aim of the study, this approach enabled us to include seminal papers on stakeholder analysis. Also, the stakeholders who took part in the consensus building exercise were mostly frontline implementers, and higher-level actors were unable to participate despite repeated efforts to reach them. However, as noted above, the involvement of frontline implementers ultimately strengthened the framework refinement by representing crucial bottom-up perspectives during the development process, while insights from higher-level policy makers were captured during key informant interviews from international and national stakeholders.

## Conclusion

While there is a wealth of theories, guidelines and approaches, empirical works providing details on how stakeholder characteristics are assessed remain scarce. We offer an adapted framework for stakeholder analysis that builds on key advances in the field and has been shown to be applicable to health policy implementation research in an LMIC context. The paper presents practical guidance on how to develop the framework domains and its specific characteristics, emphasising the importance of revisiting how complex concepts such as knowledge, interest, power and position have been defined and operationalised in stakeholder analysis studies.

While the framework was developed in the context of the Philippine health system, it is likely to be highly relevant to researchers conducting stakeholder analyses in other LMIC contexts. This is especially important for comparisons of stakeholders across countries, which require consistency in the definition of concepts, domains, indicators and scoring. Our experience emphasises the need for researchers conducting stakeholder analyses to include details and accounts of how they have operationalised and assessed the concepts, as they seek to arrive at an overall understanding of the diverse ways in which actors relate and interact with each other to shape and influence policy processes.
