# Supplementary material for: Enhancing the use of stakeholder analysis for policy implementation research: towards a novel framing and operationalised measures
Source: BMJ Glob Health. 2020 Nov 6;5(11):e002661. doi: 10.1136/bmjgh-2020-002661 (PMC7651378; doi:10.1136/bmjgh-2020-002661)
Supplement: Supplementary data [file bmjgh-2020-002661supp001.pdf]

APPENDIX 1: Preferred Reporting Items for Systematic reviews and Meta-Analyses extension for Scoping Reviews (PRISMA-ScR) Checklist

SECTION	ITEM	PRISMA-ScR CHECKLIST ITEM	Reported on Section/Paragraph
**TITLE**			
Title	1	Identify the report as a scoping review.	First section of methods
**ABSTRACT**			
Structured summary	2	Provide a structured summary that includes (as applicable): background, objectives, eligibility criteria, sources of evidence, charting methods, results, and conclusions that relate to the review questions and objectives.	Under Methods section, Scoping Review sub-section, Paragraphs 1 to 4
**INTRODUCTION**			
Rationale	3	Describe the rationale for the review in the context of what is already known. Explain why the review questions/objectives lend themselves to a scoping review approach.	Under Introduction section, Paragraphs 7 and 8
Objectives	4	Provide an explicit statement of the questions and objectives being addressed with reference to their key elements (e.g., population or participants, concepts, and context) or other relevant key elements used to conceptualize the review questions and/or objectives.	Under Methods, Scoping Review sub-section, Paragraphs 1 to 3
**METHODS**			
Protocol and registration	5	Indicate whether a review protocol exists; state if and where it can be accessed (e.g., a Web address); and if available, provide registration information, including the registration number.	Not applicable, details of scoping review process were incorporated in the manuscript, the review protocol has not been registered given the nature of the review
Eligibility criteria	6	Specify characteristics of the sources of evidence used as eligibility criteria (e.g., years considered, language, and publication status), and provide a rationale.	Under Methods, Scoping Review sub-section, Paragraphs 2 and 3, Figure 2
Information sources*	7	Describe all information sources in the search (e.g., databases with dates of coverage and contact with authors to identify additional sources), as well as the date the most recent search was executed.	Under Methods, Scoping Review sub-section, Paragraphs 2 and 3. Table 2 and Appendix 2 also provides a complete list of information sources included in the search
Search	8	Present the full electronic search strategy for at least 1 database, including any limits used, such that it could be repeated.	Under Methods, Scoping Review sub-section,

SECTION	ITEM	PRISMA-ScR CHECKLIST ITEM	Reported on Section/Paragraph
			Paragraphs 2 and 3 and Figure 2
Selection of sources of evidence†	9	State the process for selecting sources of evidence (i.e., screening and eligibility) included in the scoping review.	Under Methods, Scoping Review sub-section, Paragraphs 2 and 3
Data charting process‡	10	Describe the methods of charting data from the included sources of evidence (e.g., calibrated forms or forms that have been tested by the team before their use, and whether data charting was done independently or in duplicate) and any processes for obtaining and confirming data from investigators.	Under Methods, Scoping Review sub-section, Paragraph 4
Data items	11	List and define all variables for which data were sought and any assumptions and simplifications made.	Under Methods, Scoping Review sub-section, Paragraph 4
Critical appraisal of individual sources of evidence§	12	If done, provide a rationale for conducting a critical appraisal of included sources of evidence; describe the methods used and how this information was used in any data synthesis (if appropriate).	Not applicable, the literature sources were appraised for appropriateness.
Synthesis of results	13	Describe the methods of handling and summarizing the data that were charted.	Under Methods, Scoping Review sub-section, Paragraph 4
**RESULTS**			
Selection of sources of evidence	14	Give numbers of sources of evidence screened, assessed for eligibility, and included in the review, with reasons for exclusions at each stage, ideally using a flow diagram.	Under Methods, Scoping Review sub-section, Figure 2
Characteristics of sources of evidence	15	For each source of evidence, present characteristics for which data were charted and provide the citations.	Under Results section, Scoping Review sub-section, Paragraphs 1 to 7, Tables 2 and 3
Critical appraisal within sources of evidence	16	If done, present data on critical appraisal of included sources of evidence (see item 12).	Not applicable, Item 12 was not appropriate in this review
Results of individual sources of evidence	17	For each included source of evidence, present the relevant data that were charted that relate to the review questions and objectives.	Under Results section, Scoping Review sub-section, Tables 2 and 3
Synthesis of results	18	Summarize and/or present the charting results as they relate to the review questions and objectives.	Under Results section, Scoping Review sub-section, Paragraphs 1 to 7, Tables 2 and 3

SECTION	ITEM	PRISMA-ScR CHECKLIST ITEM	Reported on Section/Paragraph
**DISCUSSION**			
Summary of evidence	19	Summarize the main results (including an overview of concepts, themes, and types of evidence available), link to the review questions and objectives, and consider the relevance to key groups.	Under Discussion section, Paragraph 2
Limitations	20	Discuss the limitations of the scoping review process.	Under Discussion section, Paragraphs 7 and 9
Conclusions	21	Provide a general interpretation of the results with respect to the review questions and objectives, as well as potential implications and/or next steps.	Under Conclusion, Paragraphs 1 and 2
**FUNDING**			
Funding	22	Describe sources of funding for the included sources of evidence, as well as sources of funding for the scoping review. Describe the role of the funders of the scoping review.	Funding source for the study included in the Acknowledgement section.

Checklist from: Tricco AC, Lillie E, Zarin W, O'Brien KK, Colquhoun H, Levac D, et al. PRISMA Extension for Scoping Reviews (PRISMAScR): Checklist and Explanation. Ann Intern Med. 2018;169:467–473. doi: 10.7326/M18-0850.

Balane MA, *et al. BMJ Global Health* 2020; 5:e002661. doi: 10.1136/bmjgh-2020-002661

APPENDIX 2: Stakeholder analysis studies included in scoping review

1	Abiiro GA, McIntyre D. Universal financial protection through National Health Insurance: a stakeholder analysis of the proposed one-time premium payment policy in Ghana. Health Policy Plan [Internet]. 2013 May 1 [cited 2019 Jul 6];28(3):263–78. Available from: https://academic.oup.com/heapol/article-lookup/doi/10.1093/heapol/czs059
2	Ancker S, Rechel B. HIV/AIDS policy-making in Kyrgyzstan: a stakeholder analysis. Health Policy Plan [Internet]. 2015 Feb 1 [cited 2019 Jul 11];30(1):8–18. Available from: https://academic.oup.com/heapol/article-lookup/doi/10.1093/heapol/czt092
3	Behzadifar, M. *et al.* (2019) 'Hepatitis C virus-related policy-making in Iran: a stakeholder and social network analysis', *Health Research Policy and Systems*, 17(42). doi: 10.1186/s12961-019-0442-1.
4	Bryson, J. M., Patton, M. Q. and Bowman, R. A. (2011) 'Working with evaluation stakeholders: A rationale, step-wise approach and toolkit', *Evaluation and Program Planning*. Pergamon, 34(1), pp. 1–12. doi: 10.1016/J.EVALPROGPLAN.2010.07.001.
5	Caniato M, Vaccari M, Visvanathan C, Zurbrügg C. Using social network and stakeholder analysis to help evaluate infectious waste management: A step towards a holistic assessment. Waste Manag [Internet]. 2014 May 1 [cited 2019 Jul 6];34(5):938–51. Available from: https://www.sciencedirect.com/science/article/pii/S0956053X14000634?via%3Dihub
6	Durham J, Warner M, Phengsavanh A, Sychareun V, Vongxay V, Rickart K. Stakeholder Analysis of Community Distribution of Misoprostol in Lao PDR: A Qualitative Study. PLoS One [Internet]. 2016 [cited 2019 Jul 11];11(9):e0162154. Available from: http://www.ncbi.nlm.nih.gov/pubmed/27631089
7	Fischer SE, Strandberg-Larsen M. Power and Agenda-Setting in Tanzanian Health Policy: An Analysis of Stakeholder Perspectives. Int J Heal Policy Manag [Internet]. 2016 [cited 2019 Jul 10];5(6):355–63. Available from: http://ijhpm.com
8	Franco-Trigo L, Marqués-Sánchez P, Tudball J, Benrimoj SI, Martínez-Martínez F, Sabater-Hernández D. Collaborative health service planning: A stakeholder analysis with social network analysis to develop a community pharmacy service. Res Soc Adm Pharm [Internet]. 2019 May 21 [cited 2019 Jul 11]; Available from: https://www.sciencedirect.com/science/article/pii/S1551741118308040?via%3Dihub
9	Gil, A. *et al.* (2010) 'Alcohol policy in a Russian region: a stakeholder analysis', *The European Journal of Public Health*, 20(5), pp. 588–594. doi: 10.1093/eurpub/ckq030.
10	Gilson L, Erasmus E, Borghi J, Macha J, Kamuzora P, Mtei G. Using stakeholder analysis to support moves towards universal coverage: lessons from the SHIELD project. Health Policy Plan [Internet]. 2012 Mar 1

	[cited 2019 Apr 29];27(suppl 1):i64–76. Available from: https://academic.oup.com/heapol/article-lookup/doi/10.1093/heapol/czs007
11	Haidari AM, Zaidi S, Gul R. Prospects for the sustainability of delivering the Basic Package of Health Services in Afghanistan: a stakeholder analysis يف إيتاء احلزمة األساسية من اخلدمات الصحية يف أفغانستان: حتليل لألطراف املعنية آفاق ضامن االستمرار [Internet]. Vol. 20. 2014 [cited 2019 Jul 11]. Available from: http://applications.emro.who.int/EMHJ/V20/05/EMHJ_2014_20_5_300_308.pdf
12	Heydari M, Seyedin H, Jafari M, Dehnavieh R. Stakeholder analysis of Iran's health insurance system. J Educ Health Promot [Internet]. 2018 [cited 2019 Jul 5];7:135. Available from: http://www.ncbi.nlm.nih.gov/pubmed/30505863
13	Knai, C., McKee, M. and Pudule, I. (2011) 'Soft drinks and obesity in Latvia: a stakeholder analysis', *The European Journal of Public Health*. Narnia, 21(3), pp. 295–299. doi: 10.1093/eurpub/ckq062.
14	Namazzi G, Suzanne KN, Peter W, John B, Olico O, Katharine AA, et al. Stakeholder analysis for a maternal and newborn health project in Eastern Uganda [Internet]. 2013 [cited 2019 Jul 6]. Available from: http://www.biomedcentral.com/1471-2393/13/58
15	Reyes-Alcázar, V. *et al.* (2012) 'Stakeholder Analysis: The Andalusian Agency for Healthcare quality case', *The Health Care Manager*, 31(4), pp. 365–374. doi: 10.1097/HCM.0b013e31826fe373.
16	Schang, L., Thomson, S. and Czypionka, T. (2016) 'Explaining differences in stakeholder take up of disease management programmes: A comparative analysis of policy implementation in Austria and Germany', *Health Policy*, 120(3), pp. 281–292. doi: 10.1016/j.healthpol.2016.02.002.

APPENDIX 3: Topic guide for interviews

1. What policies and initiatives to address CVD are you aware of? Which ones are key?
2. Have you heard of PhilPEN? How did you hear about it?
3. What are the key features of PhilPEN? What are the key features of other policies you mentioned?
4. On the basis of everything discussed, how would you describe your organisation's level of knowledge about PhilPEN? About cardiovascular disease control policies?
    - o No or minimum knowledge
    - o General knowledge
    - o Extensive knowledge
5. Would you say that cardiovascular disease control is part of your organisation's main mission and goals? Please explain.
6. What are the priorities of your organisation for the next three years?
    - Probe on the extent to which cardiovascular disease control and PhilPEN is relevant to the organisation's priorities. How are they linked?
7. How do you think the implementation of PhilPEN (other core policy) has affected/will affect your organisation?
    - Did the implementation of PhilPEN and other CVD policies provide opportunities for the organisation? Do you see these opportunities as short term or long term?
    - Did the implementation of PhilPEN cause any disadvantages to your organisation? (An example can be cost in terms of organisation's resources)
8. Based on the discussions above, how would you rate your organisation's level of interest in implementing the policy?
    - o No or minimum interest
    - o Limited interest
    - o General interest
    - o High interest
    - o Primary interest
9. Which partners do you usually work with in the implementation of PhilPEN or CVD control, and in what way?
    - Do you work mainly with partners in CVD/NCD field or also with others, working in PHC?
    - Is your organisation a member of any alliance or coalition?
    - What kind of initiatives are these? What are the outcomes of these initiatives? (Ex. Strategic plan etc)
    - Does your organisation lead or have a substantive contribution to any initiatives to convene different stakeholders to respond to CVD? Tell us a bit more about its purpose, actions and achievements?
10. Among the partners you mentioned, and including your own organisation, which do you think are the important actors that can make decisions on how the policy will be implemented? Please explain why you think they are so.
    - Which among the partners develop guidelines and procedures for policy implementation?
    - I would like to understand the relationship between the national government and local government when it comes to the implementation of

PhilPEN and other CVD control policies. Can you describe an interaction or event that describes how good or bad this relationship is?
- How do you (or would you) engage with bottom up initiatives, ex. ideas or initiatives by patient groups, public figures or population representatives?

11. How does the implementation of the policy get funded? Which partners are involved in funding implementation of PhilPEN? Which partners fund cardiovascular disease control initiatives in general?
    - Are these funding sources available only for a certain period of time (ex. 2 to 3 years) or secure for the long term?
    - How does your organisation ensure sustainability of resources and implementation efforts?
12. Among the partners involved in implementation, and including your own organisation, who do you think have the technical knowledge and information about PhilPEN implementation? About cardiovascular disease control?
13. We talked about where your organisation stands, but having all these in mind, what is your organisation's attitude towards PhilPEN and other policies we discussed? What actions have your organisation taken to demonstrate support or opposition for the policy?
    - Extent to which they support/oppose.
        - Supporter
        - Moderate Supporter
        - Neutral
        - Moderate Opponent
        - Opponent
14. Have you encountered strong support or opposition from any stakeholder in the implementation of PhilPEN and other CVD control policies? Please explain how this support or opposition was demonstrated.
15. What do you think are the challenges of implementing PhilPEN and/or CVD control policies?
16. What recommendation can you provide to improve PhilPEN and/or CVD control implementation?
